# Supplementary material for: Repurposing Atovaquone as a Therapeutic against Acute Myeloid Leukemia (AML): Combination with Conventional Chemotherapy Is Feasible and Well Tolerated
Source: Cancers (Basel). 2023 Feb 20;15(4):1344. doi: 10.3390/cancers15041344 (PMC9954468; doi:10.3390/cancers15041344)

Supplementary Figure S1

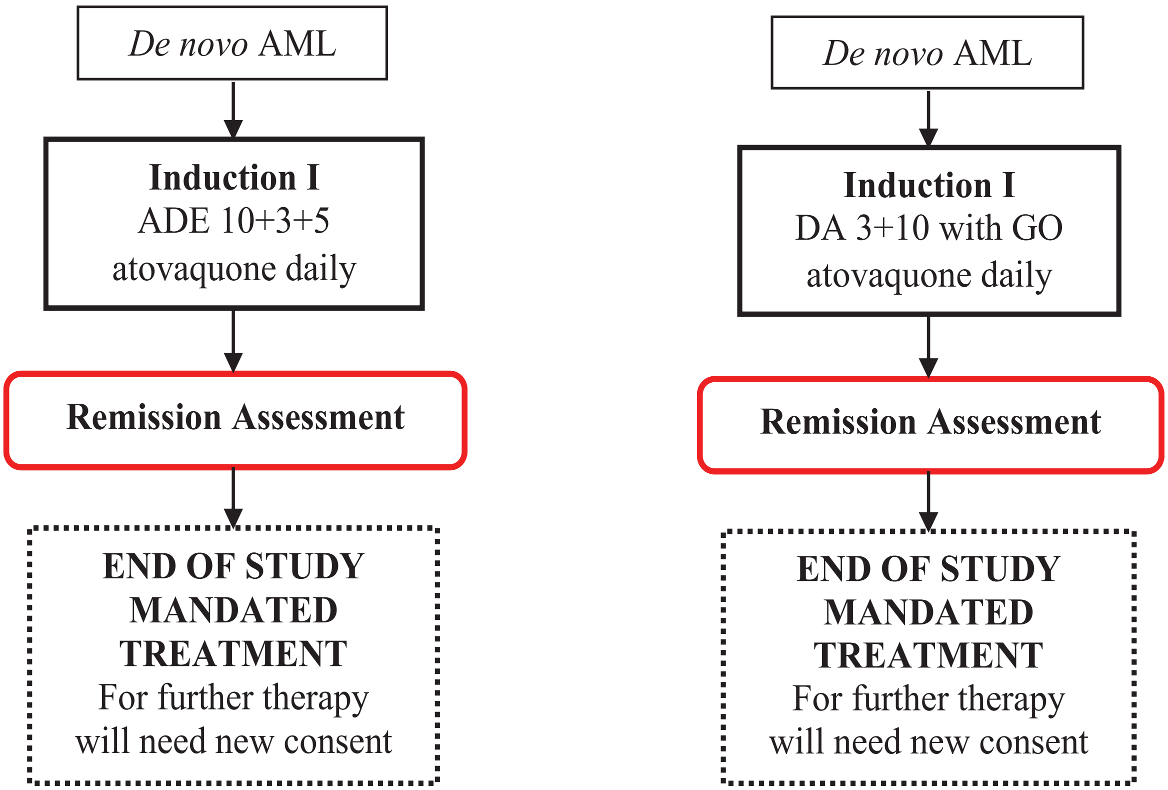

Supplementary Figure S2

A

Patient Diary
Name: ____________________________ **Page** ____
DOB: ____________________________

Atovaquone dosage*: ____________ **mg =** ____________ **ml**

Cycle day	Date Due	Route of Administration	Response to Palatability Question**	Date and time given and initials/Comments

* Please notate any dosage changes due to weight change
****Ease of Administration Question: Based on its taste, smell, and how it felt in the mouth, how easy or difficult was it for you/your child to take this medication today: (Very easy=5, Easy=4, Neither easy or difficult=3, Difficult=2, Very difficult=1).**

B

ATACC AML [redacted] Version Date: 01/29/2019
Version Number: 1.04

APPENDIX 5
Patient Diary
Name: [redacted] **Page** 1
DOB: [redacted]

Atovaquone dosage*: 1500 **mg =** 10 **ml**

Cycle day	Date Due	Route of Administration	Response to Palatability Question**	Date and time given and initials/Comments
6	7/8/19	oral syringe	4	7/8/19 6:35pm Chinese food ate 2 sour patch kids then took medicine
7	7/9/19	oral syringe	1	7/9/19 6:38pm - burger fries ate 2 sour patch kids + took - started to gag/sick
8	7/10/19	oral syringe	5	7/10/19 6:45 had Subway
9	7/11/19	oral syringe	3	7/11/19 - too a few sips of water after got down had Subway & sour patch kids
10	7/12/19	oral syringe	3	7/12/19 6:40pm had hamburger/fries took 2 sour patch green
11	7/13/19	oral syringe	3	7/13/19 7:00pm Pepperoni Pizza - 2 sour patch kids + water
12	7/14/19	oral syringe	4	7/14/19 6:30pm - Hamburger 2 sourpatch + water
13	7/15/19	oral syringe	5	7/15/19 7:56pm - Pizza rolls - 2 sour patch kids + water
14	7/16/19	oral syringe	4	7/16/18 8:18pm - Tacos, fruit jerky, B-day Cake - 2 sour patch kids + water
15	7/17/19	oral syringe	5	7/17/19 7:03pm - Hamburger + FF - 2 sour patch kids + water
16	7/18/19	oral syringe	5	7/18/19 7:12pm pizza, cinnamon sticks 2 sour patch kids + water
17	7/19/19	oral syringe	4	7/19/19 8:05pm Cheese burger, FF, Sprite 2 sourpatch kids water
18	7/20/19	oral syringe	3	7/20/19 6:45pm Subway 12in. Lemonade 2 sourpatch kids water
19	7/21/19	oral syringe	4	7/21/19 6:57pm Schlotzsky's Sandwich 2 sour patch kids water
20	7/22/19	oral syringe	3	7/22/19 7:05pm pizza rolls 2 sour patch kids water
21	7/23/19	oral syringe	3	7/23/19 7:32pm Cheeseburger skittles followed by water
22	7/24/19	oral syringe	3	7/24/19 8:20 Crawfish etouffe 2 sour patch kids water
23	7/25/19	oral syringe	5	7/25/19 8:25 Tacos 2 sour patch kids water
24	7/26/19	oral syringe	5	7/26/19 8:20 fajitas 2 sour patch kids water

* Please notate any dosage changes due to weight change
****Palatability Question: Based on its taste, smell, and how it felt in the mouth, how easy or difficult was it for you/your child to take this medication today: (Very easy=5, Easy=4, Neither easy or difficult=3, Difficult=2, Very difficult=1).**

Supplementary Figure S3

A

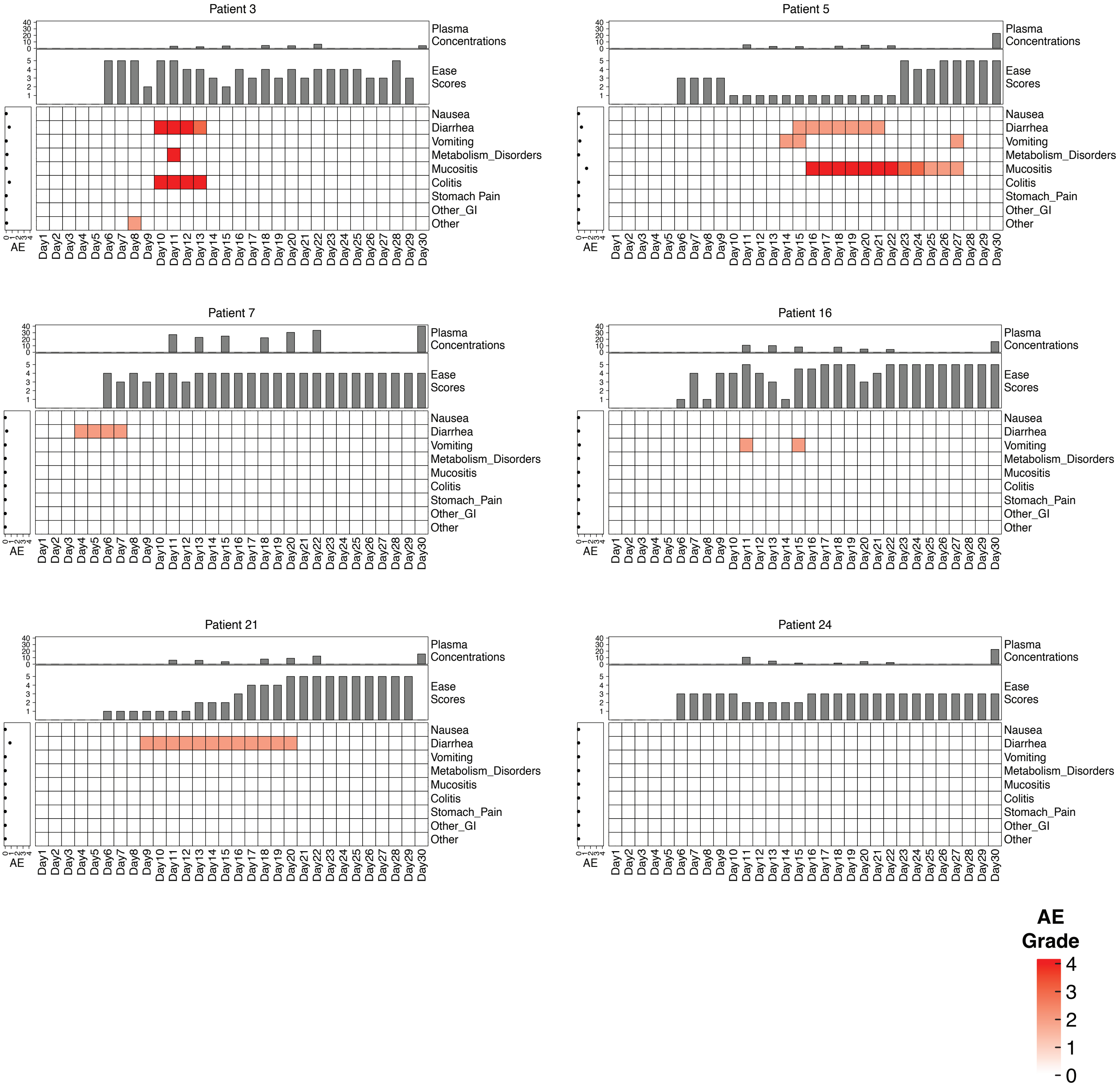

Supplementary Figure S3

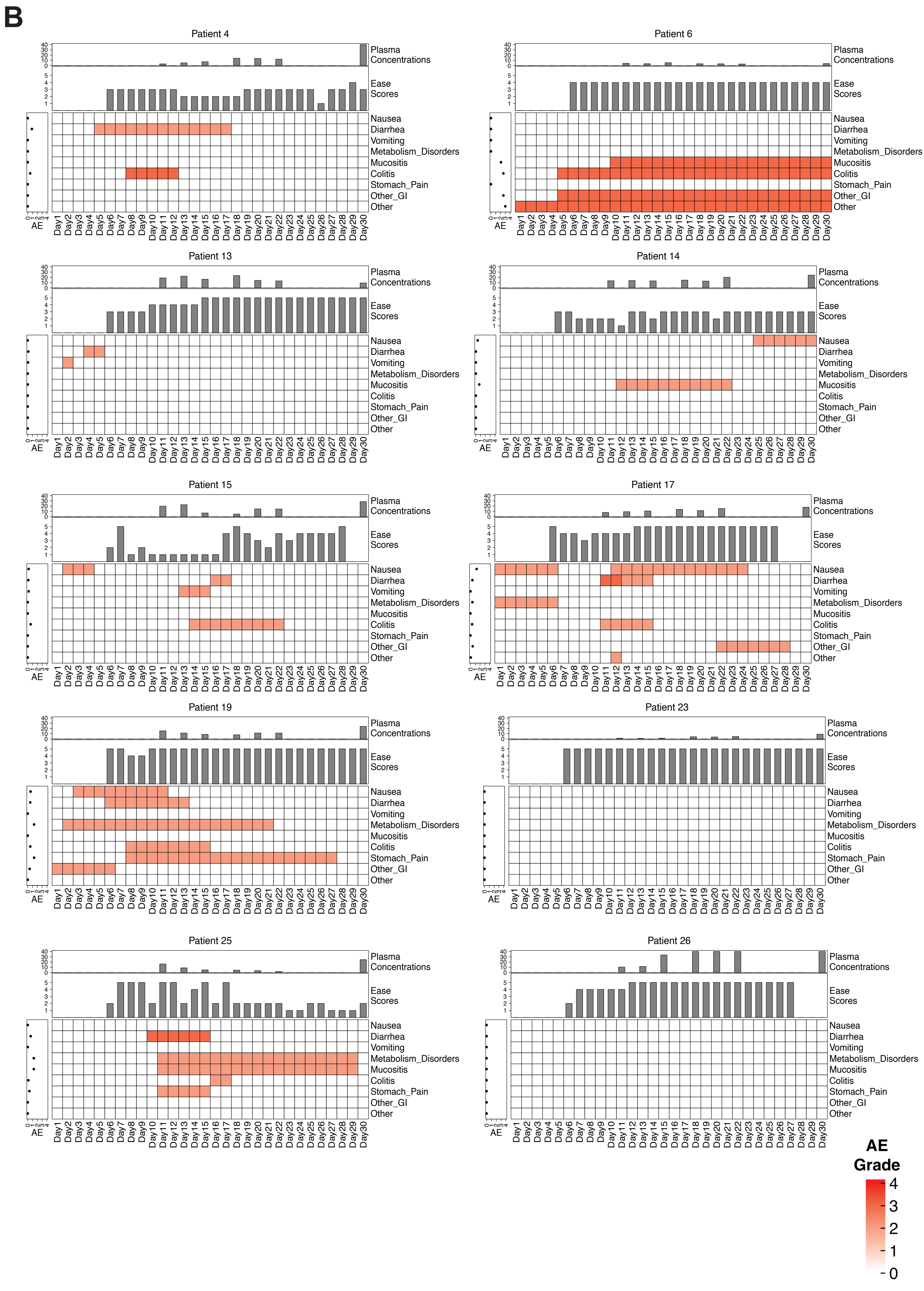

Supplementary Figure S3

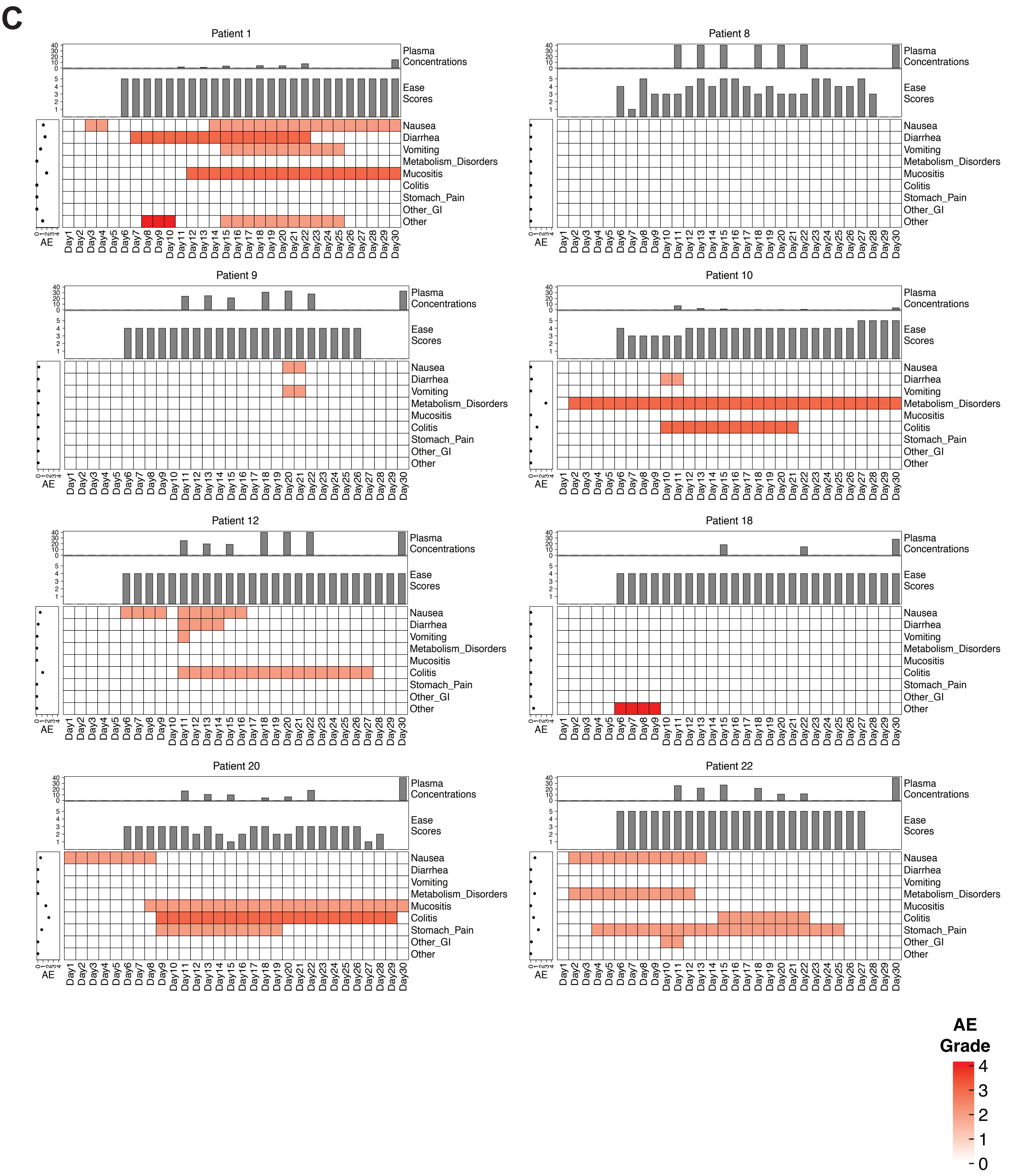

Supplementary Figure S4

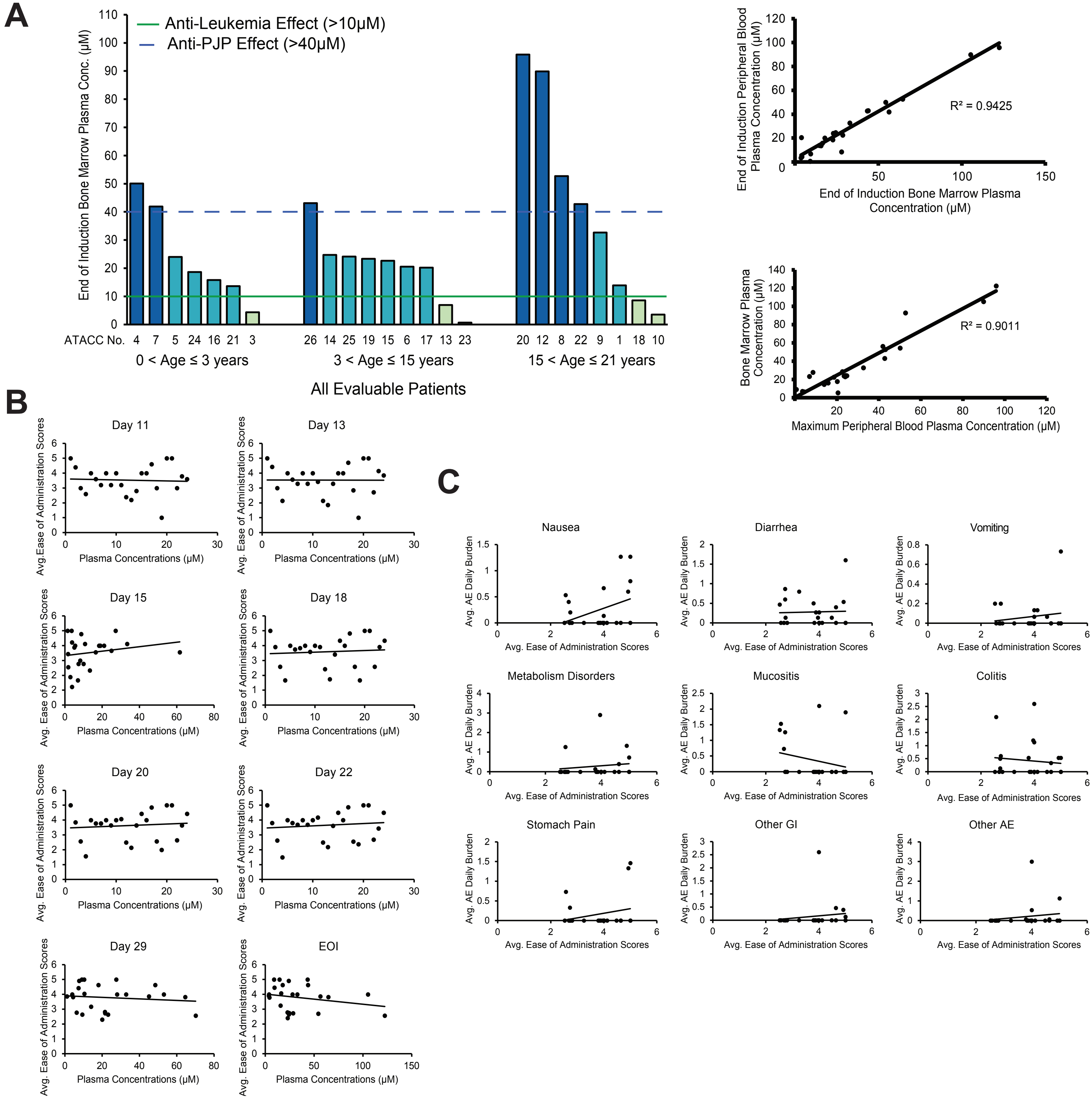

D

Average Ease of Administration Scores vs Average AE Burden

	Nausea	Diarrhea	Vomiting	Metabolic Disorders	Mucositis	Colitis	Stomach Pain	Other GI	Other
Pearson Coefficient (r)	0.383	0.033	0.174	0.134	-0.228	-0.108	0.242	0.143	0.166
P-value	0.064	0.877	0.417	0.533	0.285	0.616	0.254	0.505	0.439

Average Ease of Administration Scores vs Plasma Concentrations

	Day 11	Day 13	Day 15	Day 18	Day 20	Day 22	Day 29	EOI
Pearson Coefficient (r)	-0.035	-0.055	0.194	0.230	0.085	0.155	-0.117	-0.238
P-value	0.875	0.804	0.363	0.291	0.705	0.480	0.587	0.263

Supplementary Figure SF5

A

All Patient Samples in Media+FBS

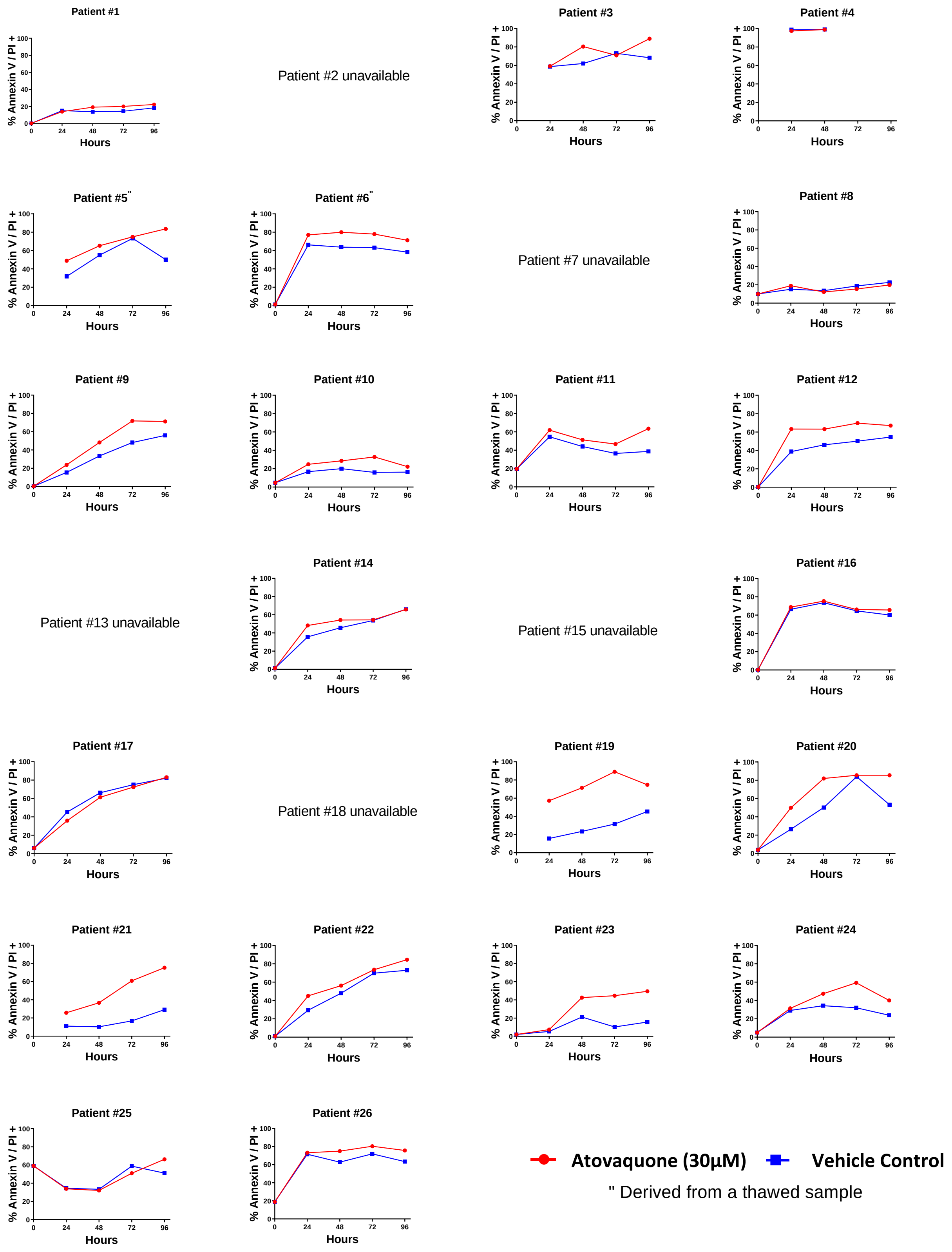

Supplementary Figure SF5

B

All Patient Samples in CM

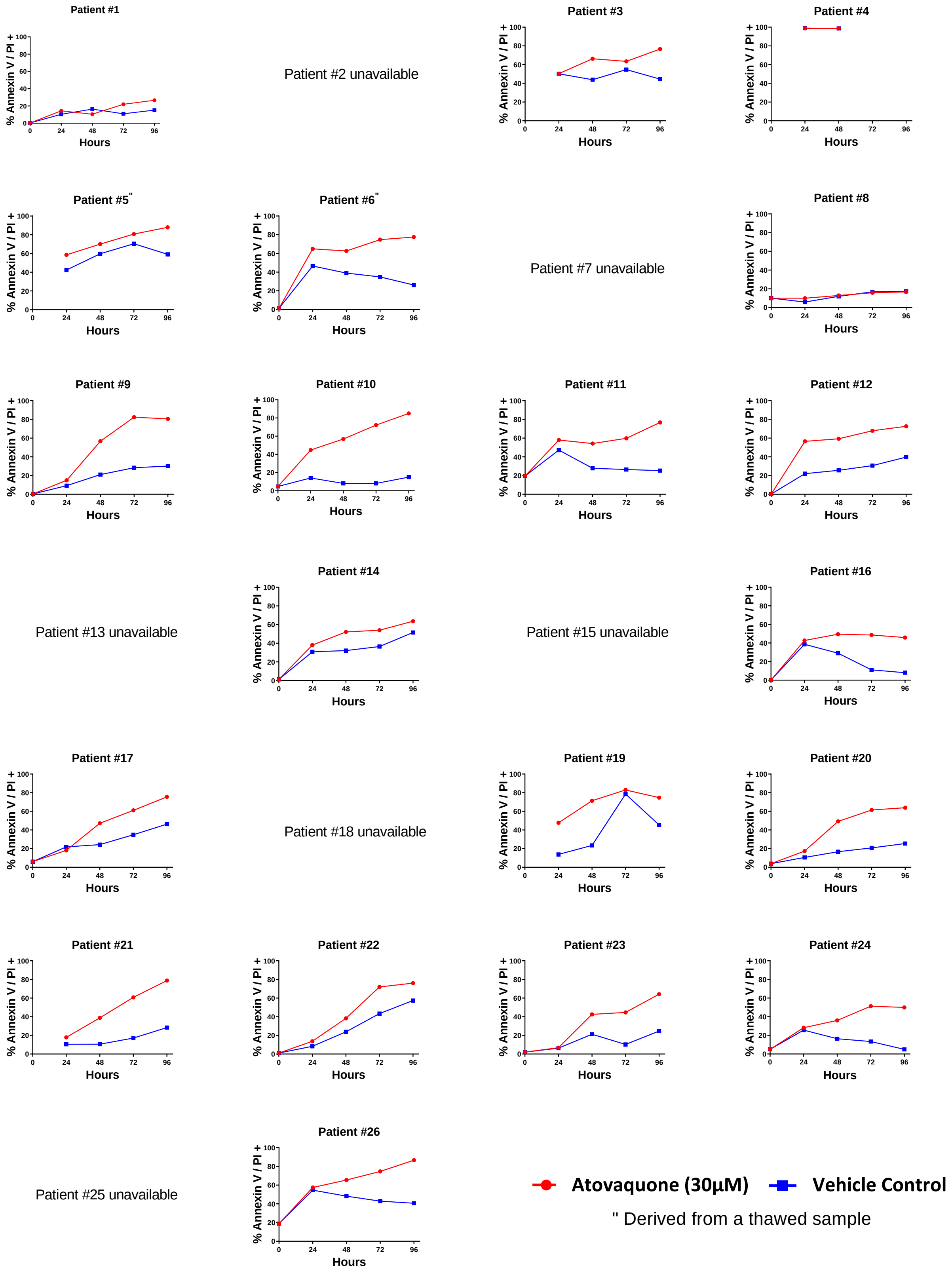

Supplementary Figure SF5

C

All Patient Samples in TW

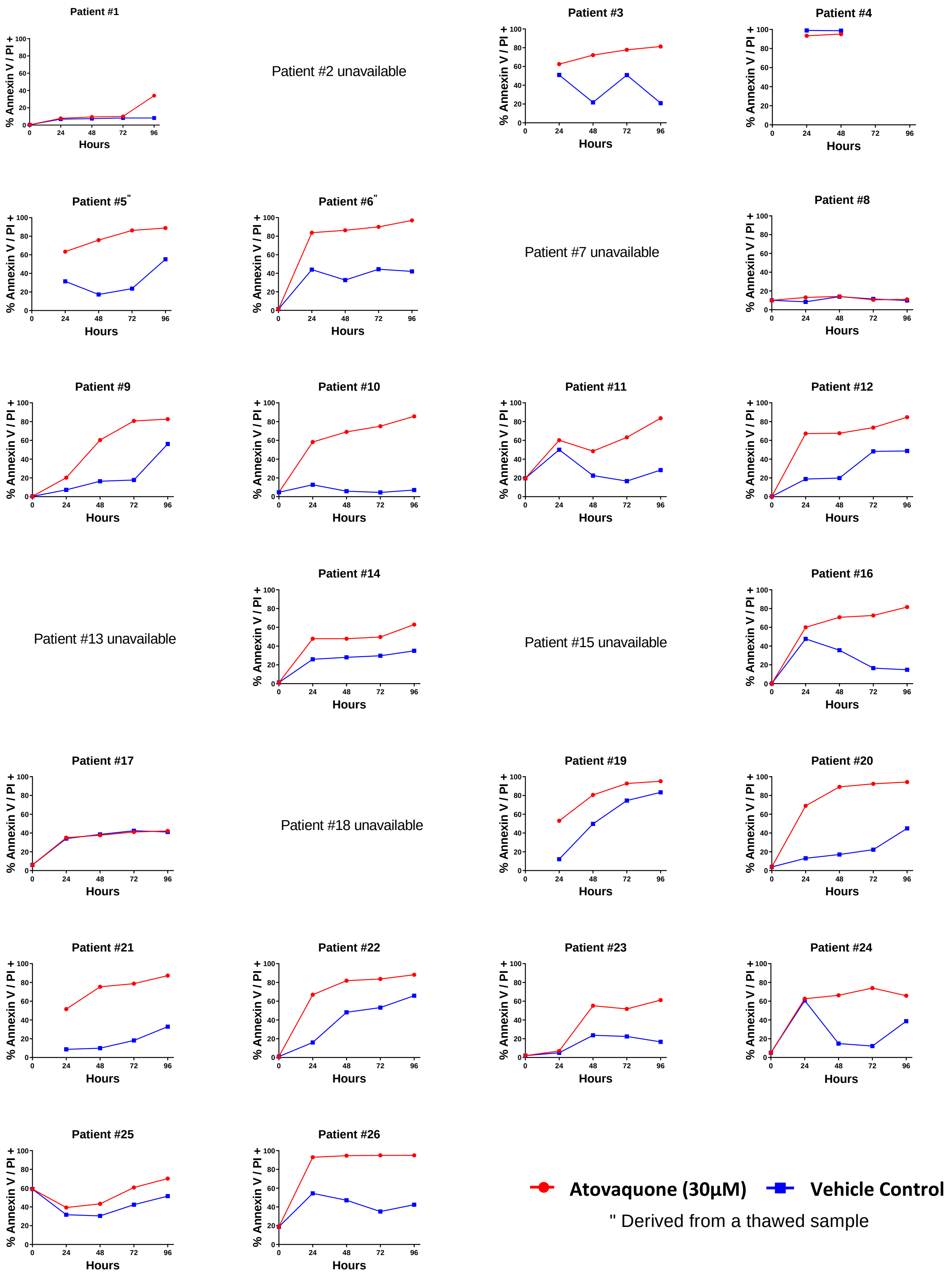

Supplementary Figure SF6

Fresh vs. Thawed Samples

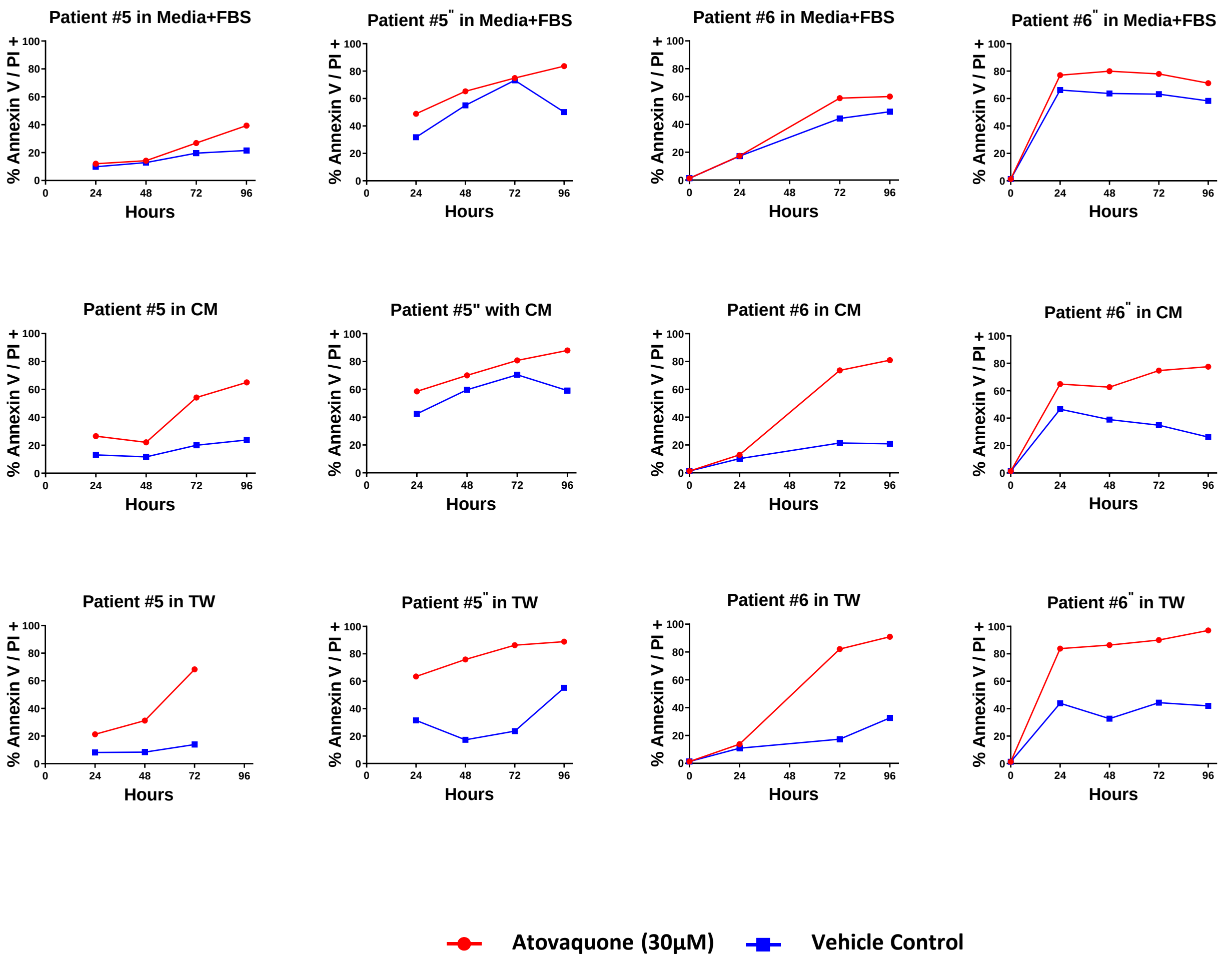

" Derived from a thawed sample

Supplementary Figure SF7

A

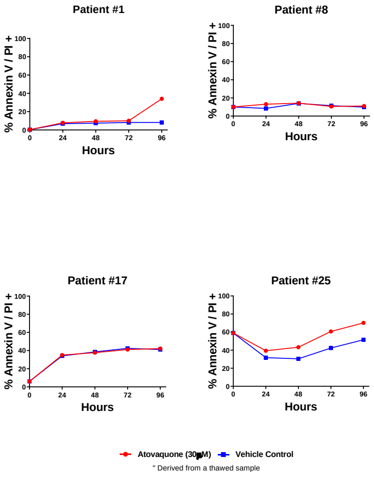

C

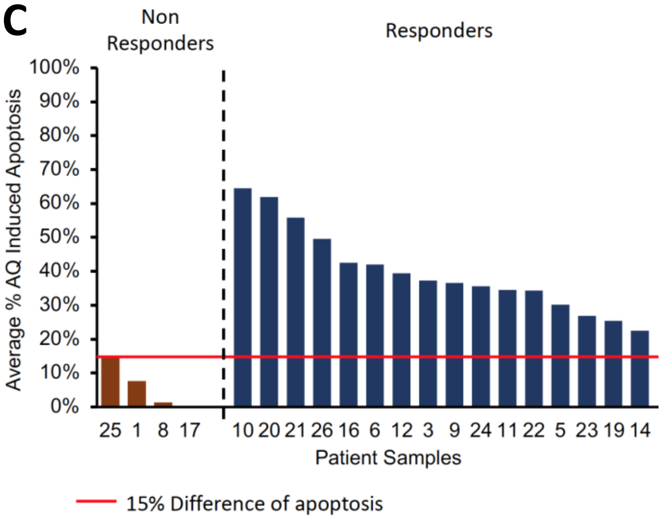

B

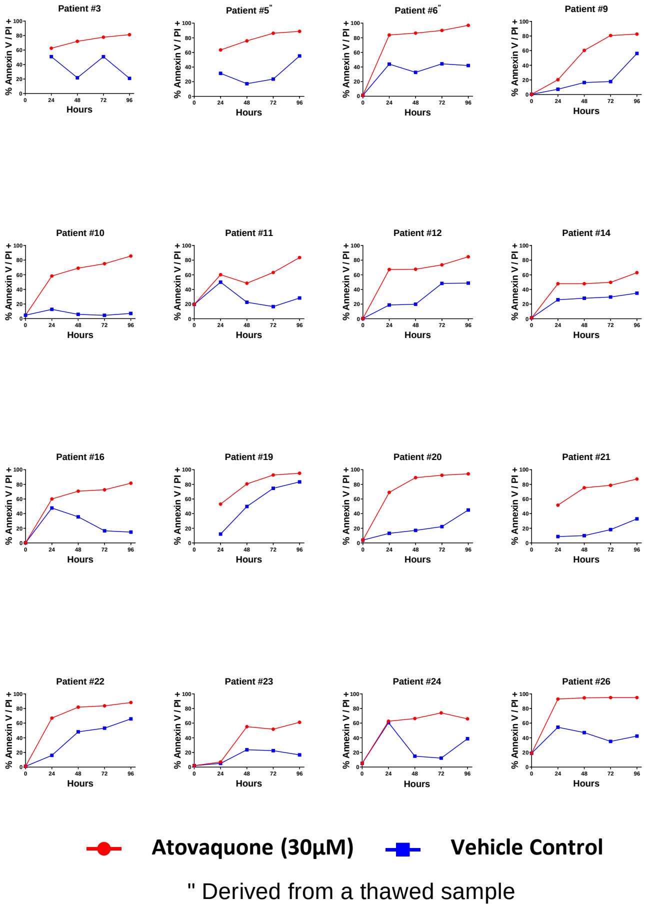

Supplementary Figure SF8

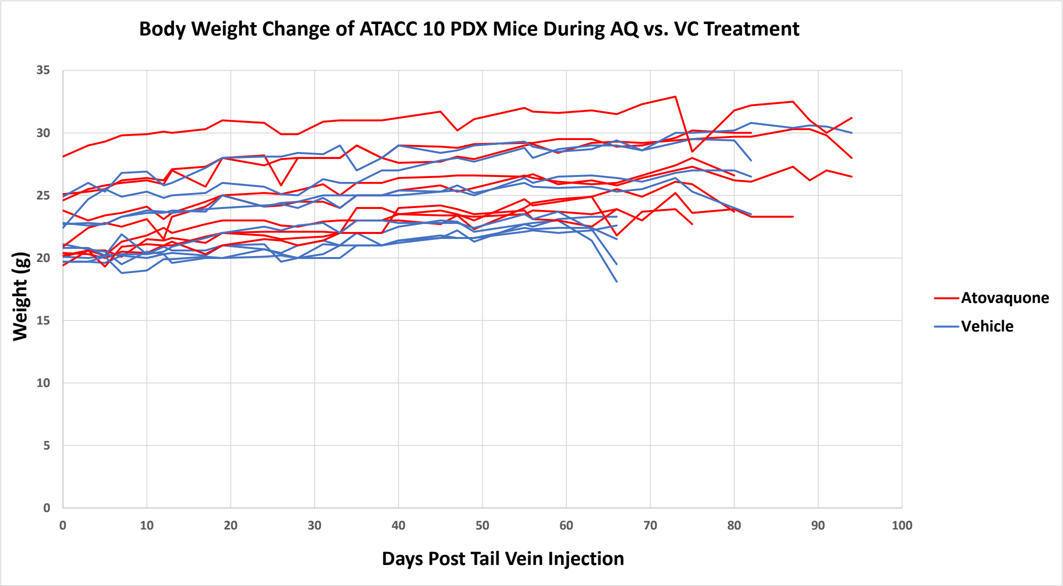

Supplementary Figure SF9

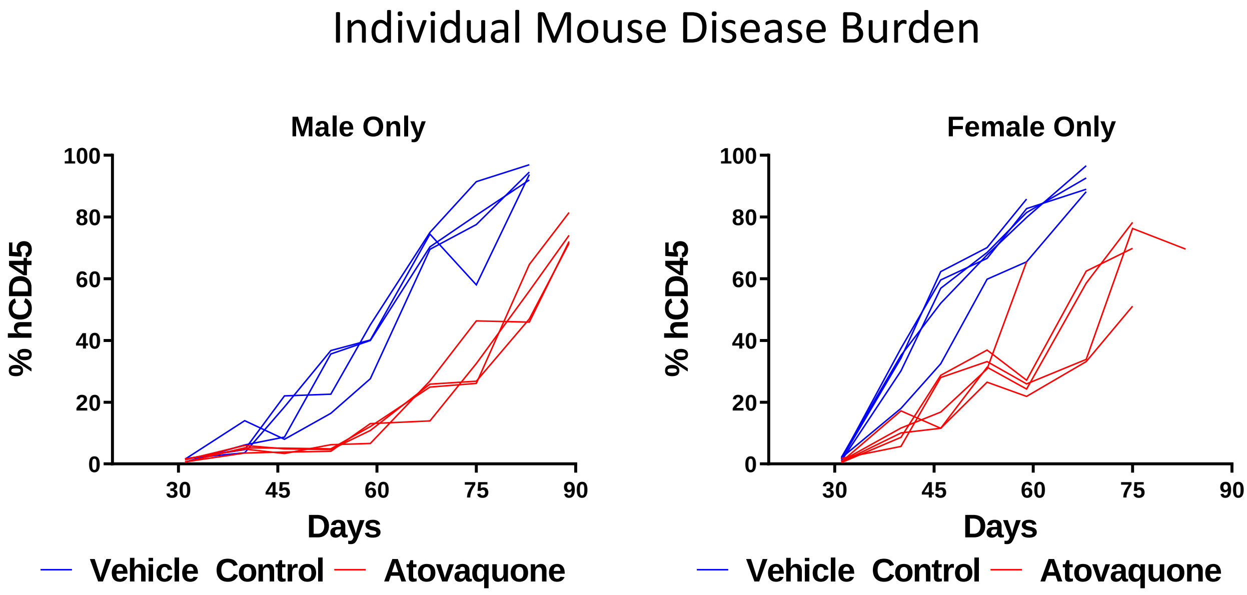

Supplementary Figure SF10

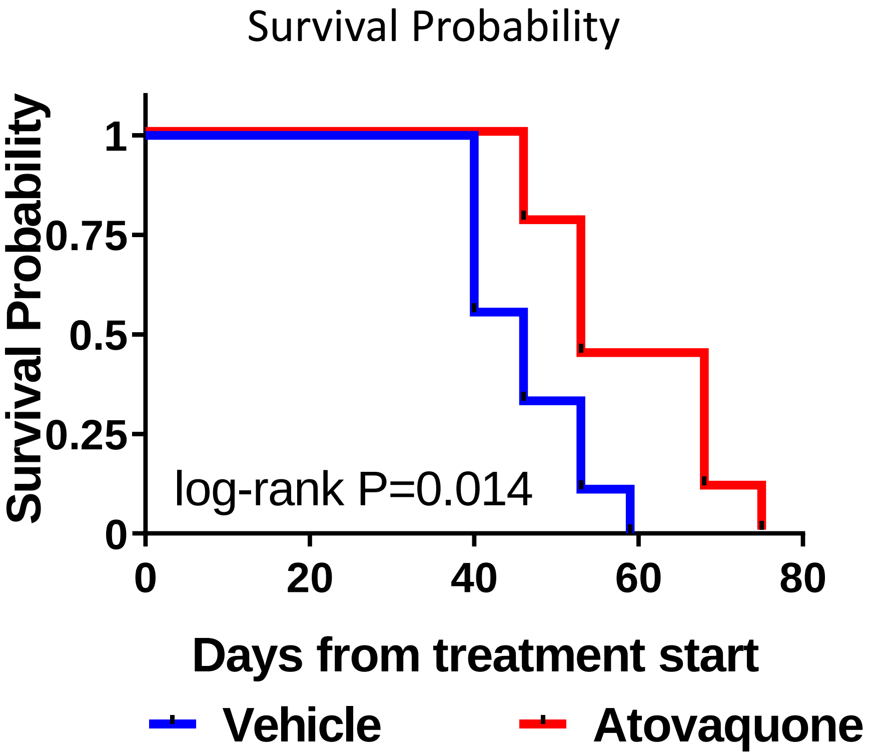

Supplementary Figure SF11

OCR for All Study Patients

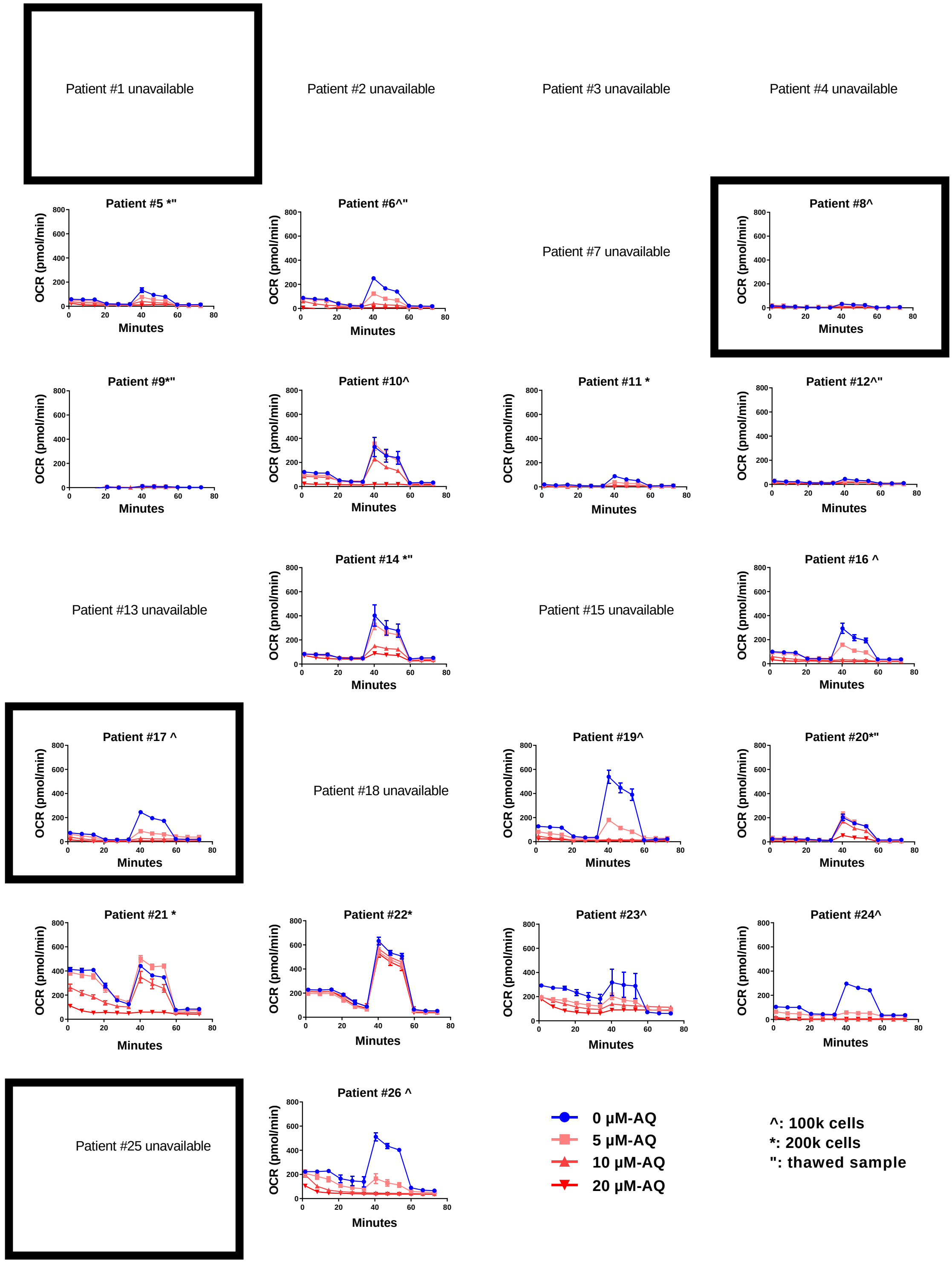

Supplementary Figure SF12

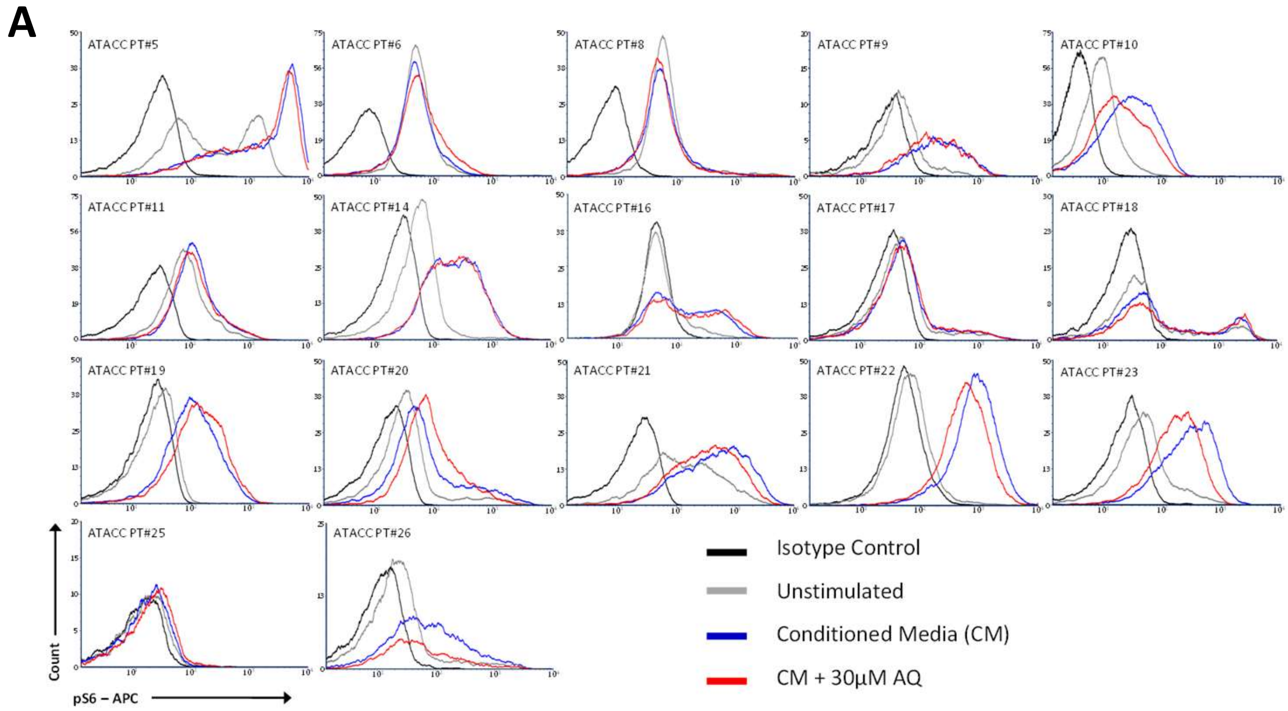

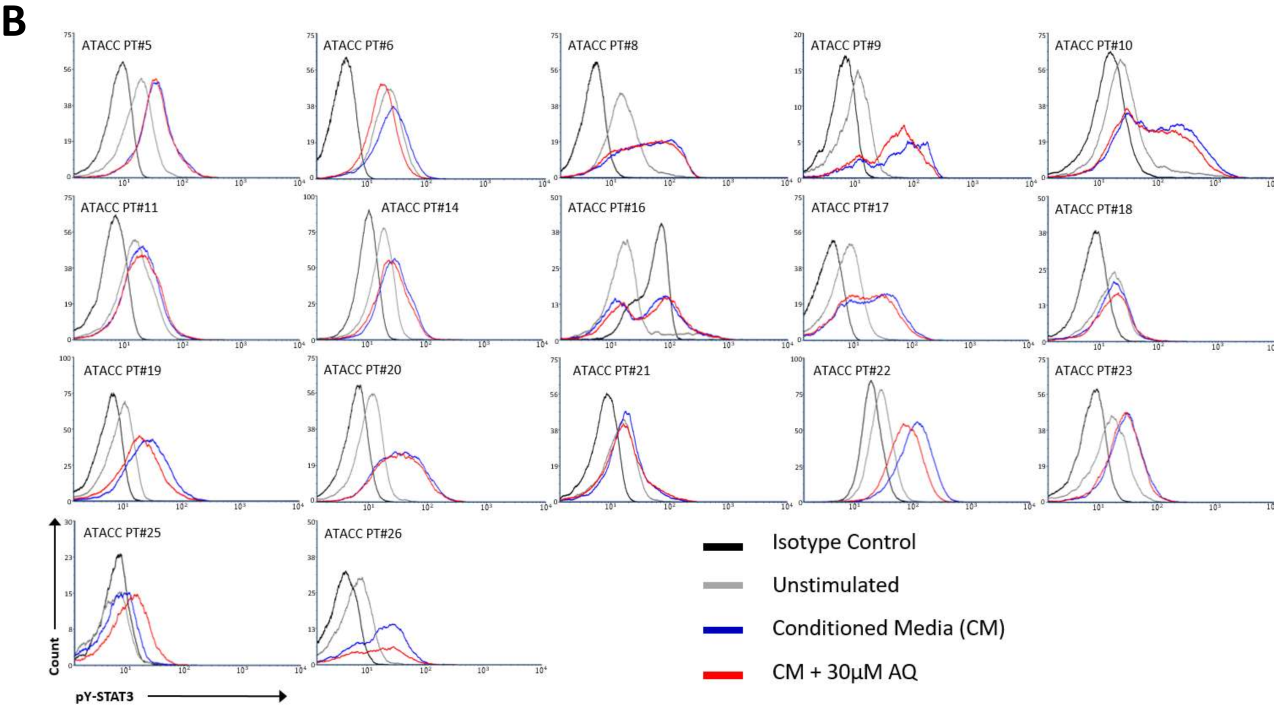

Supplementary Figure SF12

C

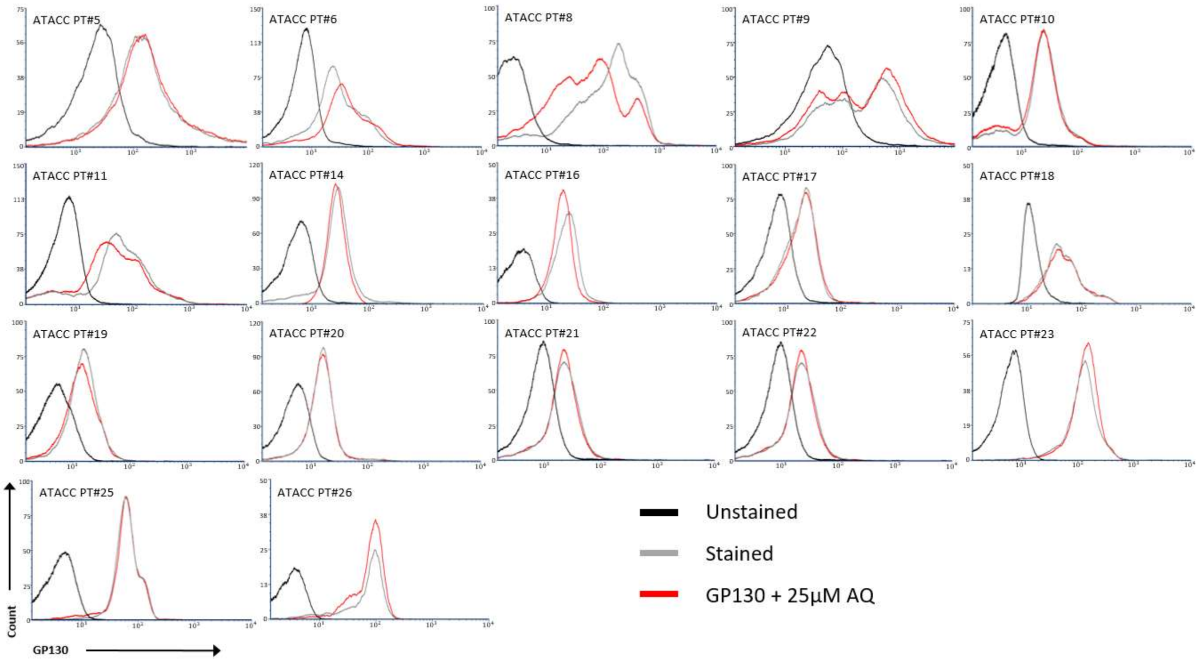

D

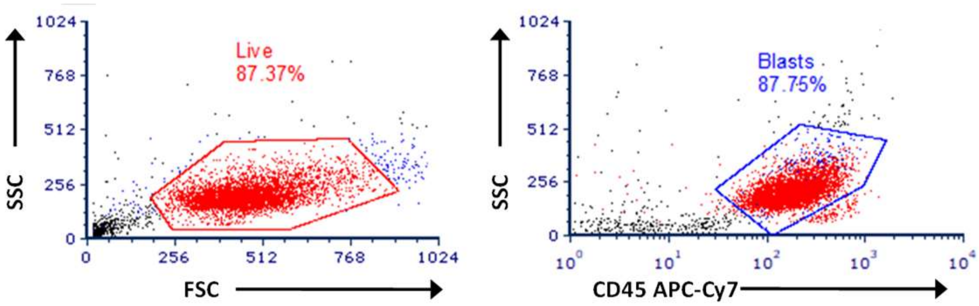

Supplement: Supplementary file 1 [file cancers-15-01344-s001.zip › ATACC Manuscript Supplementary Figures with modified SF12 2-10-23.pdf]
