# Supplementary material for: Repurposing Atovaquone as a Therapeutic against Acute Myeloid Leukemia (AML): Combination with Conventional Chemotherapy Is Feasible and Well Tolerated
Source: Cancers (Basel). 2023 Feb 20;15(4):1344. doi: 10.3390/cancers15041344 (PMC9954468; doi:10.3390/cancers15041344)
Supplement: Supplementary file 1 [file cancers-15-01344-s001.zip › ATACC manuscript supplementary tables 7-8-22 retitled as S1-S3.pptx]

## Slide 1
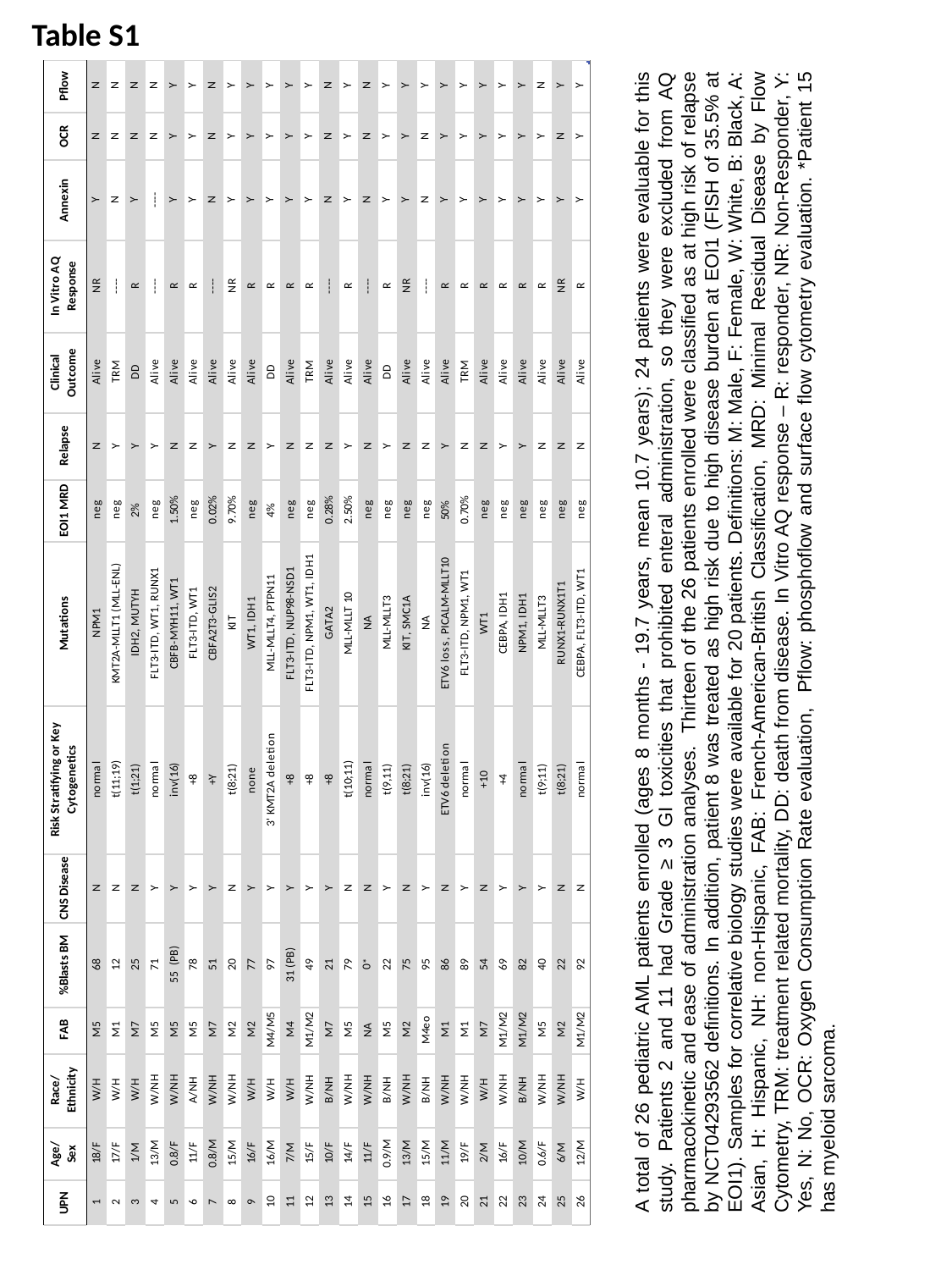

Table S1
A total of 26 pediatric AML patients enrolled (ages 8 months - 19.7 years, mean 10.7 years); 24 patients were evaluable for this study. Patients 2 and 11 had Grade ≥ 3 GI toxicities that prohibited enteral administration, so they were excluded from AQ pharmacokinetic and ease of administration analyses. Thirteen of the 26 patients enrolled were classified as at high risk of relapse by NCT04293562 definitions. In addition, patient 8 was treated as high risk due to high disease burden at EOI1 (FISH of 35.5% at EOI1). Samples for correlative biology studies were available for 20 patients. Definitions: M: Male, F: Female, W: White, B: Black, A: Asian, H: Hispanic, NH: non-Hispanic, FAB: French-American-British Classification, MRD: Minimal Residual Disease by Flow Cytometry, TRM: treatment related mortality, DD: death from disease. In Vitro AQ response – R: responder, NR: Non-Responder, Y: Yes, N: No, OCR: Oxygen Consumption Rate evaluation, Pflow: phosphoflow and surface flow cytometry evaluation. *Patient 15 has myeloid sarcoma.

## Slide 2
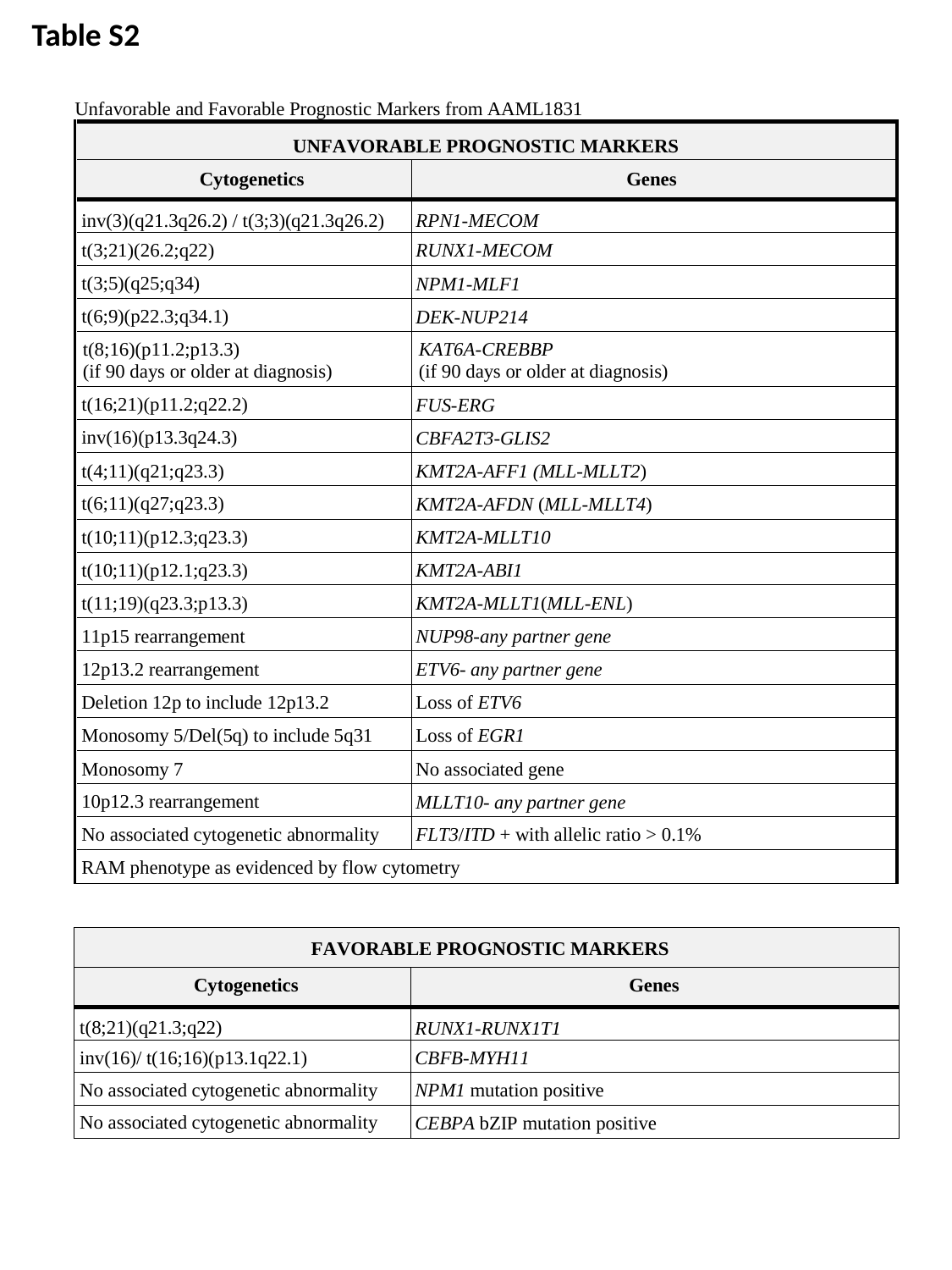

Table S2

## Slide 3
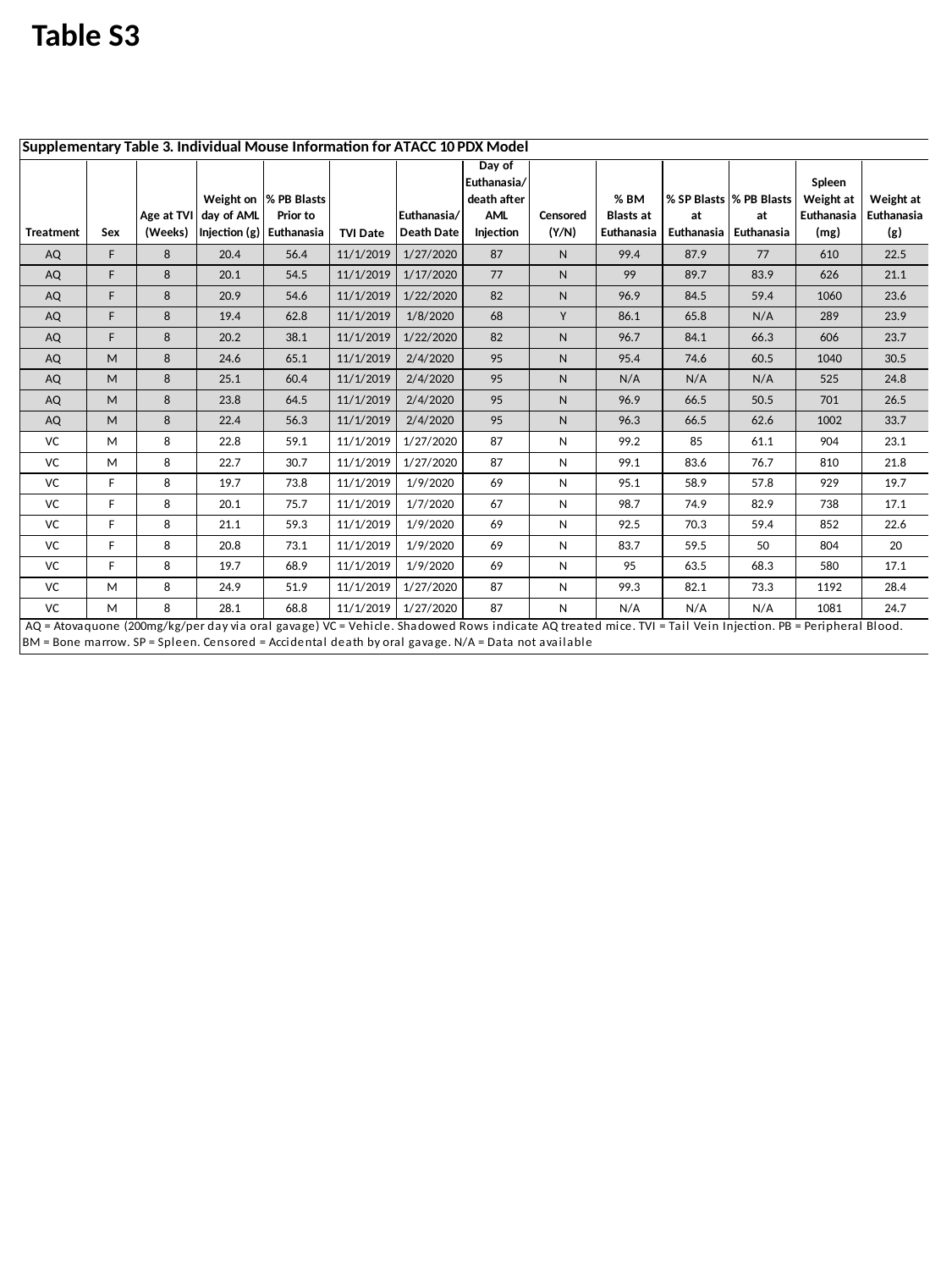

Table S3
